# Codon optimality has minimal effect on determining translation efficiency in mycobacterium tuberculosis

**DOI:** 10.1038/s41598-022-27164-0

**Published:** 2023-01-09

**Authors:** Smitha Soman, Somdeb Chattopadhyay, Siya Ram, Vinay Kumar Nandicoori, G. Aneeshkumar Arimbasseri

**Affiliations:** 1 School of Biotechnology, Gautam Buddha University, Gautam Budh Nagar, Greater Noida, Uttar Pradesh India; 2 Molecular Genetics Laboratory, National Institute of Immunology, Aruna Asaf Ali Marg, New Delhi, 110067 India; 3 School of Sciences, Indira Gandhi National Open University, Maidan Garhi, New Delhi, 110068 India; 4 Centre for Cellular and Molecular Biology, Habsiguda, Uppal Road, Hyderabad, Telangana 500007 India

**Keywords:** Biochemistry, Computational biology and bioinformatics

## Abstract

*Mycobacterium tuberculosis* (Mtb) is a slow-growing, intracellular pathogen that exhibits a high GC-rich genome. Several factors, including the GC content of the genome, influence the evolution of specific codon usage biases in genomes. As a result, the Mtb genome exhibits strong biases for amino acid usage and codon usage. Codon usage of mRNAs affects several aspects of translation, including accuracy, efficiency, and protein folding. Here we address the effect of codon usage biases in determining the translation efficiency of mRNAs in Mtb. Unlike most commonly studied organisms, Mtb carries a single copy of each tRNA gene. However, we show that the relative levels of tRNAs in the Mtb tRNA pool vary by an order of magnitude. Our results show that the codons decoded by the abundant tRNAs indeed show higher adaptability. Moreover, there is a general positive correlation between genomic codon usage and the tRNA adaptability of codons (TAc). We further estimated the optimality of the codon and mRNAs by considering both the TAc and the tRNA demand. These measures did not show any correlation with mRNA abundance and translation efficiency. There was no correlation between tRNA adaptability and ribosome pausing as well. Taken together, we conclude that the translation machinery, and the tRNA pool of an organism, co-evolve with the codon usage to optimize the translation efficiency of an organism. Thus the deleterious effect of maladapted codons is not pronounced.

## Introduction

Species-specific codon bias is a well-documented phenomenon and is a significant determinant of the successful expression of heterologous genes. Even within a species, highly abundant mRNAs appear to have distinct codon usage^1^. The ability of intracellular pathogens such as viruses to express their genes in the host cells depends on viral gene codon usage^2–4^. Several pieces of evidence suggest viruses such as HIV can modulate the host tRNA pool to aid the translation of viral mRNAs^5,6^. Studies in mammalian systems have identified differences in the cellular tRNA pools in a tissue-specific manner, which may aid translation in a tissue-specific manner^7^.

Chemical modifications on tRNA bases are known to be important for the pathogenicity of various microbial pathogens. These changes in tRNAs are especially required during adaptation to stress conditions elicited by the host organism. Methylation of C32 of tRNA^Met^CAT and tRNA^Trp^CCA is essential for the oxidative stress response of Pseudomonas aeruginosa. Deletion of the gene that adds this methylation makes this bacteria sensitive to hydrogen peroxide^8^. In Shigella flexneri, the tRNA modification ms2i6A is essential for expression of virulence genes^9^. Lethal infection of mice with E. coli leads to changes in the modification patterns of tRNAs^10^. Interestingly, changes in tRNA modifications have also been shown to alter amino acid uptake by E. coli^11^. One of the mechanisms by which tRNA modifications alter the translation efficiency is by changing the decoding efficiency of tRNAs. In such a scenario, the codon usage of the mRNAs will have an effect on the translation efficiency of mRNAs. In Mycobacterium tuberculosis, one of the leading causes of mortality due to infectious diseases, the role of codon usage or tRNAs in the determination of translation efficiency is unknown. Interestingly, tRNA modifications have been shown to play an important role in the adaptation of Mtb to hypoxic conditions, which it encounters during infection^12^. The first step in understanding the role of tRNAs in the regulation of protein synthesis of Mtb is to understand the correlation between codon usage and translation efficiency.

Codon usage bias and codon efficiency have been proposed to play an important role in the regulation of translation efficiency, accuracy and folding^13–15^. Nevertheless, the role of codon usage in regulating translation efficiency within critical pathogens such as *Mycobacterium tuberculosis* (Mtb) is not well understood. Several measures have been developed to assess the relationship between codon usage and translation efficiency. The Codon adaptation index, or CAI, was initially developed by measuring the codon usage of highly abundant mRNAs in a cell^16^. The rationale was that highly abundant mRNAs should be adapted to the tRNA pool of a cell and have optimal codon usage. The mRNAs that share the codon usage pattern with the highly abundant mRNAs could be more adapted to the translational milieu of the cell.

An important aspect to consider when studying codon usage and translation efficiency is the availability of tRNAs. Ikemura et. al.^1^ had shown that the codon usage of abundant mRNAs is driven by the abundance of tRNAs in the cell. dos Reis et. al.^17^ developed another measure, the tRNA adaptation index (tAI), to estimate the adaptation of codons and mRNAs to the tRNA pool of a cell. In this method, the availability of tRNAs that decode each codon was used to estimate the adaptability of codons. The more abundant the tRNAs that decode a codon, the more adapted the codon is. Moreover, the mRNAs that carry more codons decoded by abundant tRNAs are expected to have adapted to the tRNA pool of the cell. Adaptations of these methods were later developed to understand the relationship between codon usage and translation efficiency in a species-specific manner^18,19^. However, no such attempt has been made for Mtb.

While it is appropriate to consider tRNA abundance to estimate the codon and mRNA adaptation, this model has two caveats. First, the gene copy number of tRNAs is considered a measure of tRNA abundance. For single-celled organisms such as *E. coli* and *S. cerevisiae*, the number of genes corresponds to the abundance of tRNAs. So, using gene copy numbers as a proxy for tRNA abundance is appropriate. However, organisms such as Mtb or *Plasmodium falciparum* have only one copy of gene per tRNA, making it impossible to estimate the tRNA levels from gene copy numbers. The second caveat of the original tAI algorithm is that the demand for tRNAs in the transcriptome is not considered. The abundance of mRNAs in the cell determines the demand for codons. The advantage of a highly abundant tRNA in the cell is neutralized if their demand is equally high. A modification of the original tAI algorithm was introduced, which integrates the tRNA demand into the calculation of codon adaptability^14^.

Apart from the availability of tRNAs, tRNA demand, and wobble base pairing, other factors also determine the adaptability of codons within a cell. Some of them include tRNA modifications and tRNA aminoacylation. In this study, we asked if codon adaptability, as a function of tRNA abundance and tRNA demand, plays any role in the translation efficiency of mRNAs in Mtb. To address this, we estimated the adaptability codons in Mtb based on the tRNA abundance. We included the demand for anticodons in the calculation besides the tRNA abundance for the calculation of codon optimality. Further, we estimated the mRNA optimality score and found that only a few mRNAs in Mtb have suboptimal codon usage. Moreover, the translation efficiency of these mRNAs was not low, suggesting that the codon adaptability determined by tRNA abundance and demand is not the determining factor for translation efficiency in Mtb.

## Results

### Mtb tRNA pool exhibit a wide range of tRNA levels

**Figure 1:**
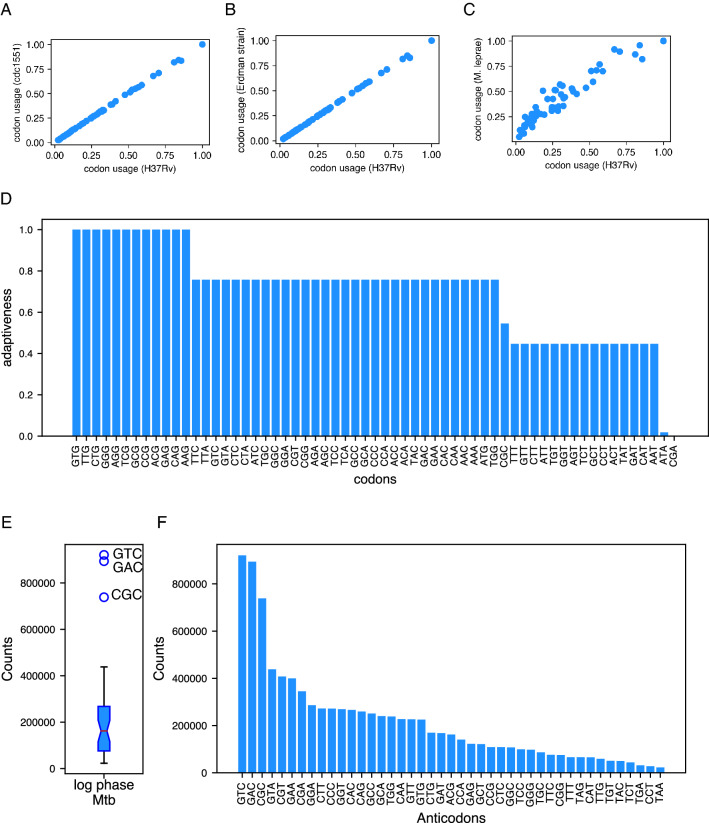
Mtb tRNA pool exhibit wide variability in the abundance of tRNAs. (**A–C**) Scatterplots showing genomic codon usage values of H37Rv and CDC1551 (**A**), H37Rv and Erdman strain (**B**), and H37Rv and M. leprae (**C**). (**D**) TAc values calculated using the gene copy numbers. (**E**) A box plot showing normalized read counts obtained from a tRNA-HySeq experiment done with log phase Mtb. (**F**) Normalized read counts for each anticodon depicted as a barplot.

First, we analysed if different strains of Mtb exhibits similar codon usage pattern. To address this, we calculated the genomic codon usage of three Mtb strains—H37Rv, CDC1551, and Erdman strain. Figure 1A and B shows that the codon usage is nearly identical for these three strains. Moreover, we compared the codon usage of H37Rv strain of Mtb with a closely related slow growing pathogen, M. leprae. Interestingly, the codon usage pattern of these two organism shows a strong positive correlation (Fig. 1C). Rest of the experiments and analyses described are done with the H37Rv strain of Mtb, which is a standard laboratory reference strain.

The first step in defining the adaptability of codons to the tRNA pool in a cell is to estimate the relative abundance of tRNAs that are available to decode each codon. Previous studies have used the tRNA gene copy number as a reliable proxy for tRNA abundance in organisms such as yeast and *E. coli*. However, this approach was unsuccessful in mammalian systems such as mice and humans with much higher numbers of tRNA genes (> 400 genes)^17^. Silencing many tRNA genes by structural changes in chromatin, which enables tissue-specific tRNA expression^7,20^, may be responsible for the lack of correlation between tRNA gene copy number and tRNA abundance. In Mtb, all tRNA genes are in single copies, which makes it impossible to use gene copy numbers as a proxy for tRNA abundance. Indeed, the tRNA adaptability values of codons (TAc) calculated using tRNA gene copy numbers show that wobble base pairing is the only determinant of TAc (Fig. 1D).

Next, we asked if Mtb cells exhibit a uniform abundance of tRNAs, as suggested by the gene copy numbers. To address this, we quantified the tRNA levels in the H37Rv strain by performing tRNA-HySeq^21^. We subjected total tRNAs to partial digestion, followed by adapter ligation, reverse transcription, and next-generation sequencing. The sequences obtained were analyzed for quality, mapped to a custom genome file containing Mtb tRNA gene sequences, and the reads mapping to annotated tRNA genes were counted (Fig. 1E). Unlike the assumption made from the gene copy numbers, the tRNA abundance adjudged from the tRNA-HySeq show a variability ranging an order of magnitude (Fig. 1F). tRNAAspGTC, tRNAValGAC, and tRNAAlaCGC exhibited the highest read counts, while tRNALeuTAA and tRNAIleTAT were the lowest (Fig. 1E & F).

**Figure 2:**
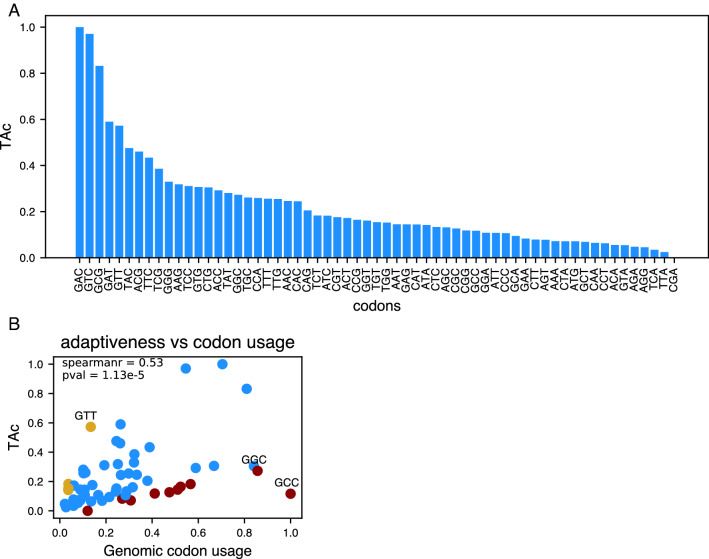
tRNA adaptability of codons in Mtb. (**A**) TAc values calculated using the tRNA abundance rather than gene counts. (**B**) A scatterplot showing the correlation between the genomic codon usage and TAc values. Spearman correlation coefficient and the p-value are given in the plot.

We used the actual tRNA abundance estimated from tRNA-HySeq to calculate the TAc for each of the codons in Mtb. As expected, the codons decoded by the three tRNAs that are highly abundant in the cell (GAC, GTC, and GCG decoded by tRNAAspGTC, tRNAAspGTC, and tRNAAlaCGC, respectively) are the codons that exhibit the highest adaptiveness (Fig. 2A). Moreover, the codons GAT and GTT decoded by tRNA^Asp^GTC and tRNA^Val^GAC by wobble base-pairing were the fourth and fifth most adapted codons (Fig. 2A). Interestingly, codon CGA, for which the only decoding tRNA is the tRNAArgACG, exhibits the least codon adaptiveness. The high penalty applied to the I-A wobble base pair could be the reason for the very low adaptiveness of the CGA codon^17^.

Highly abundant codons are considered to be more adapted to the cellular tRNA pool. To address if this is true, we directly compared the genomic usage levels of codons with the TAc. A scatter plot (Fig. 2B) suggests a positive correlation between genomic codon usage and the TAc (Spearman correlation = 0.53 and *P*-value = 1.13e-5). However, there are significant exceptions (codons marked as brown and yellow in Fig. 2B), including the codons GCC and GGC, which exhibit the highest genomic codon usage, but low levels of TAc.

### High and low abundance mRNAs exhibit similar codon usage in Mtb

**Figure 3:**
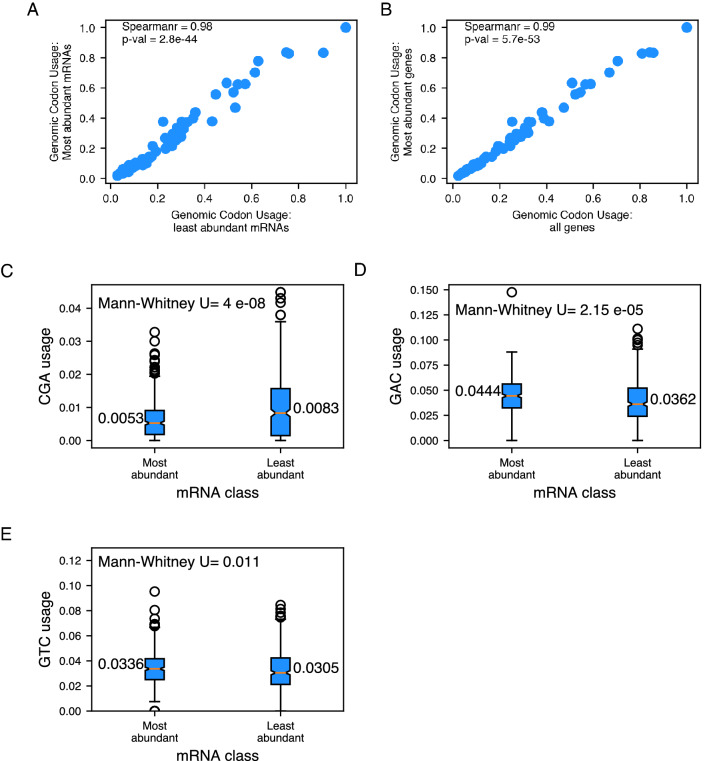
There are no drastic differences in the codon usage for most abundant and least abundant mRNAs in Mtb. (**A**, **B**) Scatterplots showing the correlation between most abundant mRNAs and least abundant mRNAs (**A**) and all mRNAs (**B**). Spearman correlation coefficient and p-values are given in the plot. (**C–E**) Comparison of usage of codons **CGA** (**C**), **GAC** (**D**), and **GTC** (**E**) between most abundant and least abundant mRNAs. *P*-value from a Mann–Whitney U test is given. Numbers on the sides of the boxes indicate the median values.

Previous studies had indicated differential codon usage between high-abundance and low-abundance mRNAs^1,16^. It was also suggested that the demand for tRNAs, determined by the mRNA abundance, is also a determinant of codon adaptability^14^. So, we addressed if the codon usage varies for different classes of mRNAs based on their abundance in Mtb. To test this, we performed RNAseq analysis of Mtb grown to log phase. After normalization of the counts, the mRNAs were divided into ten equal bins based on their read counts; each bin had 399–404 mRNAs. Comparing codon usage for all mRNAs or the least abundant mRNAs with the most abundant mRNAs revealed no marked difference (spearman r > 0.98; Fig. 3A & B). To further confirm if there is any relationship between the usage of low TAc codons and mRNA abundance, we directly compared the usage of CGA codon between the most abundant and least abundant mRNAs. The bin with the least abundant mRNAs shows a statistically significant but very small enrichment (Mann–Whitney *P*-value 4 × 10^−8^) for the CGA codon (Fig. 3C). On the other hand, the codons GAC and GTC, which show maximum adaptability values, show a slight but statistically significant enrichment among the most abundant mRNAs (Fig. 3D & E).

### tRNA adaptability does not correlate with ribosome pausing

**Figure 4:**
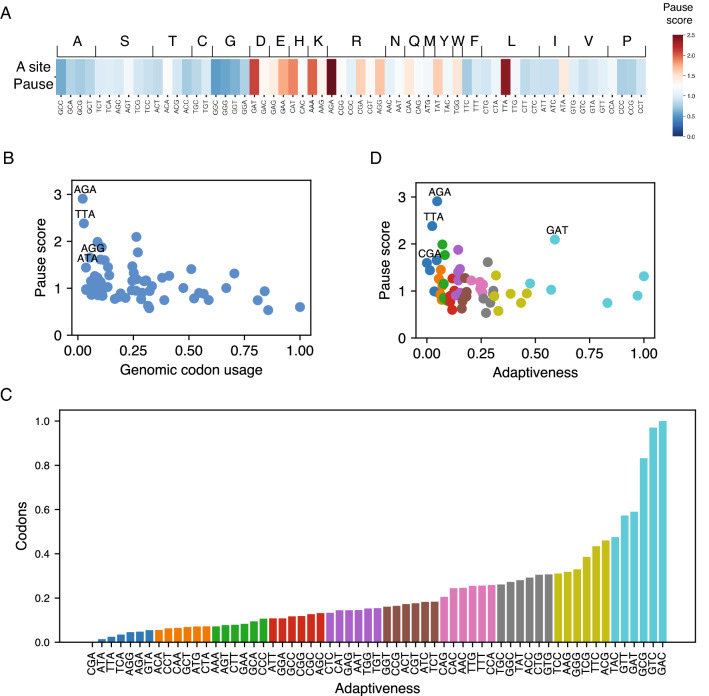
TAc does not correlate with ribosome pausing. (**A**) A heatmap showing the pause scores calculated from the riboseq data. (**B**) A scatterplot comparing pause scores with genomic codon usage. (**C**) A bar plot showing different bins of TAc values. Bins are colour coded and the same colours are used in D. (**D**) A scatterplot comparing TAc with the pause scores.

The low adaptability of codons to the tRNA pool is expected to reduce the rate at which ribosome translates that codon. As a result, suboptimal codons may lead to ribosome pausing. To understand the relationship between TAc and ribosome pausing, we performed ribosome profiling analysis of Mtb in the logarithmic growth phase^22^. This data was used to calculate codon-wise pause scores at the A site of the ribosome (Fig. 4A).

Four of the ten least used codons (AGAArg, TTALeu, ATAIle, and AGGArg) appear to have a high pause at the A site (Fig. 4A and B). However, a more intriguing observation is the higher pause on codons for charged amino acids: D, E, H, K, and R. All these amino acids except arginine have two box codons where G/C ending codons are decoded by Watson–Crick base pairing while A/T ending codons are decoded by wobble base pairing. In these cases, Despite being decoded by the same tRNA, the pause scores were higher for A- and T-ending codons compared with their G- and C-ending counterparts (Fig. 4A), indicating that the tRNA activity could be determining the decoding efficiency. A comparison of the genomic codon usage and the pause score did not show any correlation between these two measures, except that the two codons that show maximum pause also happens to be the least used codons (Fig. 4B).

Next, we addressed if Tac values show any correlation with the ribosome pausing. To visualize this relationship better, we divided the codon adaptation values into ten equal bins and color-coded these bins, as in Fig. 4C. When the adaptation values were plotted against the pause scores, we observed that the codons exhibiting the highest pause scores, AGA and TTA, exhibited low tRNA adaptability values (Fig. 4D). However, the codon CGA, which exhibits the lowest tRNA adaptability value, does not exhibit very high levels of pause (Fig. 4D). Moreover, the GAT codon for Asp, which exhibits the third-highest pause score, is among the codons with high tRNA adaptability. These results indicate that the TAc also does not play any deterministic role in ribosome pausing. There could be other factors, such as tRNA demand, tRNA modifications, and the nature of the amino acids, which play essential roles in the optimality of a codon and the dynamics of decoding.

### Codon optimality does not correlate with translation efficiency in Mtb

**Figure 5:**
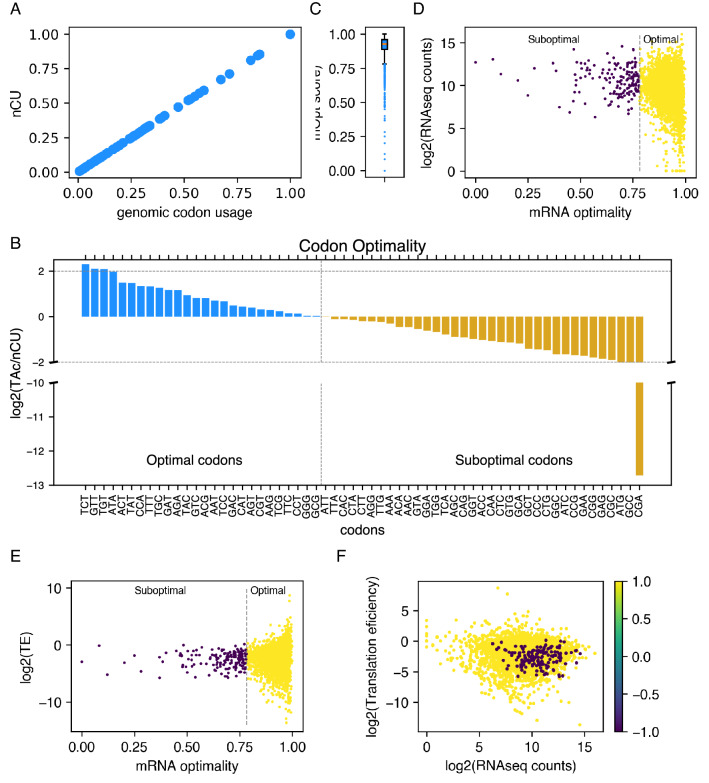
mRNA adaptability does not correlate with translation efficiency. (**A**) A scatterplot comparing nCU with genomic codon usage. (**B**) A bar plot showing the codon adaptability (cOpt) values . The golden coloured bars indicate suboptimal codons and blue bars indicate optimal codons. (**C**) A box plot showing the distribution of mRNA optimality values (mOpt). The blue dots below the bottom whisker indicate the outliers. (**D**, **E**) A scatterplot comparing the mOpt values with mRNA abundance (**D**) and translation efficiency (**E**). (**F**) A scatterplot comparing the translation efficiency and mRNA abundance with the suboptimal mRNAs identified in purple colour.

Next, we calculated the optimality of codons (cOpt) and mRNAs (mOpt) based on the tRNA pool. The cOpt values are a measure of the tRNA adaptability in relation to codon usage. If a codon is highly used in the genome, it is considered to have a high demand. Thus cOpt values represent TAc in the context of tRNA demand. Demand for tRNAs can be accurately represented only if we consider the abundance of codons in the transcriptome. To address this, we incorporated the mRNA abundance estimated from the transcriptome data into the calculation of abundance-normalized codon usage (nCU). Interestingly, the nCU values were the same as the one observed for genomic codon usage (Fig. 5A). This corroborates the observation that low-abundance and high-abundance mRNAs exhibited very similar codon usage (Fig. 3).

Any codon with a ratio of TAc to nCU higher than one is considered optimal. These ratios were log-transformed to obtain codon optimality scores (cOpt) with directionality (Fig. 5B). Any codon with a positive value is considered optimal, while any codon with a negative value is considered suboptimal (Fig. 5B). We found that only 26 codons show a positive value. The codon CGA remained the lowest adapted codon even after applying this measure. We found ten codons exhibit a log2 (ratio) lower than − 2. The codon CGA, which has the lowest TAc, shows the lowest TA/CU ratio, while GCC and GGC, which are the most abundant codons (Fig. 2B), also show a very low ratio (Fig. 5B). On the other hand, three codons (yellow in Fig. 2B) show higher TAc despite having low genomic codon usage. Interestingly, codon GTT exhibits a low codon usage but is one of the top 5 codons with high TAc (Fig. 5B).

The next question was whether the mRNAs enriched in suboptimal codons exhibit lower translation efficiency. To address this, we summed the cOpt values of each codon for each mRNA to estimate the mRNA optimality score (mOpt) (Fig. 5C). We set the cut-off values to define outliers as 1.5 times the interquartile range from Q1 (for low mOpt mRNAs; 0.782) or Q3 (for high mOpt mRNAs; 1.07). According to this calculation, 160 mRNAs show low levels of codon optimality while no mRNAs exhibit unusually high mOpt (Fig. 5C). Interestingly, an analysis comparing the abundance with the mOpt of the mRNAs revealed that the mRNAs with low codon optimality were not among the low-abundance mRNAs. Most of them show high abundance in the transcriptome of the log phase cells (Fig. 5D).

Further, we analyzed if the codon optimality of mRNAs is reflected in their translation efficiency. To address this, we estimated the translation efficiency of the mRNAs from the riboseq and RNAseq data sets using the Ribodiff package. A comparison of the translation efficiency of mRNAs and the codon optimality indicates no correlation between the codon optimality and translation efficiency of mRNAs (Fig. 5E). Figure 5F confirms that the low mOpt mRNAs are among the highly abundant mRNAs in transcriptome and exhibit average translation efficiency. These results indicate that codon adaptability, determined solely from the tRNA abundance and demand, hardly plays any role in determining the translation efficiency of mRNAs.

Finally, we conclude that the vast majority of the mRNAs in Mtb are adapted to the tRNA pool of the organism, as their translation efficiencies do not vary dramatically. Interestingly, even the mRNAs that exhibit very low levels of adaptability do not exhibit a low translation efficiency compared with other mRNAs. This suggests that codon usage may have a minimal role in determining the translation efficiency of mRNAs.

## Discussion

Identifying the determinants of translation efficiency is essential to understand the basic mechanisms of gene expression and designing synthetic biology applications. Codon usage is one factor that modulates mRNAs’ translation efficiency. The expression of heterologous mRNAs is specifically affected by the differential codon usage between different species. However, whether codon usage difference plays any role in the regulation or translation efficiency of mRNAs within a cell is still under debate^23^. Many reported methods that correlate codon usage and translation efficiency consider factors such as wobble base pairing and tRNA gene copy number^17^. In this study, we utilized the tRNA gene abundance as measured by tRNA-HySeq, tRNA demand as measured by codon abundance, and actual translation efficiency calculated using riboseq to probe the relationship between codon usage and translation efficiency in Mtb.

Here, we introduced three improvements in the estimation of mRNA optimality based on codon usage are the following. First, most algorithms used the tRNA gene number as the proxy for tRNA abundance. For organisms such as Mtb, which have single copies of each of the tRNAs, this approach will not work. Our quantitation of tRNAs in Mtb shows that the amount of tRNAs in Mtb varies by an order of magnitude and is similar to the range of tRNA abundance in mammalian cells^24^. So, it is essential to know the actual tRNA abundance to calculate the tAI of mRNAs rather than the tRNA gene copy numbers.

Second, as described in a previous study, we introduced the tRNA demand into calculating mRNA optimality^14^. Here, we confirmed that the genomic codon usage indeed represents the actual codon abundance in the transcriptome by comparing it with values obtained using the codon usage weighted for each of the mRNAs in the transcriptome. By taking the ratio of tRNA availability and the demand, we could estimate the actual adaptability of the codon to the cellular tRNA pool. A ratio greater than one indicates that the availability of tRNA is higher than the demand^14^. Using the log-transformed ratio also helped us to classify the codons as optimal and suboptimal. Aggregation of the codon optimality values of an mRNA was used to calculate mRNA optimality.

We observed that most mRNAs exhibit an mRNA optimality score between 0.78 and 1. Though there was a correlation between the TAc and genomic codon usage, no such correlation was observed between mOpt and mRNA abundance or translation efficiency. Previous attempts to understand the role of codon usage in determining the translation efficiency of mRNAs within a cell have also yielded mixed results. For example, one of the studies has shown that the codon usage in the 5' region of mRNAs plays a vital role in translation by acting as a ramp for ribosomes. Another study has shown that cell cycle-related genes exhibit higher usage of rare codons, and their expression is associated with increased total tRNAs in cell^25^. Though this study does not show a difference in the composition of the tRNA pool, an increase in the total tRNA levels may likely increase tRNAs for rare codons and aid their translation.

Another analysis of a library of clones that differ in their synonymous codon usage has revealed several log-fold differences in protein expression^26^. However, these expression levels were not associated with any predictable changes in the codon usage; instead, the effect of nucleic acid sequence differences on the secondary structure of the mRNA had a more substantial influence on the protein levels.

Moreover, a study that quantified the speed with which the ribosomes decode codons showed no difference in the decoding rate between rare codons and abundant codons^23^. They suggested that a balance between codon usage and the tRNA abundance optimizes the translation efficiency of mRNAs. Data presented here also suggest a correlation between tRNA availability and genomic codon usage. However, highly abundant codons do not necessarily mean increased translation efficiency. It is most likely that the increased usage of codons with abundant anticodons may help maintain the normal translation rates of most of the mRNAs. Even ribosome pausing did not correlate with codon usage or tRNA adaptability; instead, the chemical nature of the amino acids had a higher impact on ribosome pausing. Studies on *E. coli* have also revealed ribosome pausing on specific amino acids rather than rare codons^27^. Taken together, we conclude that most of the Mtb mRNAs are well adapted to the tRNA pool of the cell, and the tRNA availability and codon usage play a minimal role in regulating translation efficiency.

## Methods

### Media and growth conditions for M. tb

M. tb H37Rv strain was grown in 7H9 broth till OD600 was 0.4–0.6. The bacteria were harvested by rapid centrifugation, and cell pellets were physically disrupted in lysis buffer (100 µg/mL chloramphenicol, 70 mM KCl, 10 mM MgCl_2_, 10 mM Tris–HCl pH 7.4, and 5 mM CaCl_2_).

### Ribosome profiling library preparation

Bacterial lysates were cleared and digested with 4 units of S7 nuclease (Worthington Biochemical), RNase T1 (Worthington Biochemical), RNase I (Thermo Fisher Scientific), or combinations of these at room temperature for 45 min in a lysis buffer. The reaction was stopped by adding 200 units of SUPERase.In (Thermo Scientific), essentially as described (31). The stability of polysomes under these conditions was confirmed using polysome profiling.

RNA from monosome fractions of digested lysates were isolated using Trizol reagent (TaKaRa Bio) and subjected to library preparation as described. In short, RNA was size-selected using RNA oligos 25 and 35 nucleotides long as markers on a 15% TBE-Urea gel followed by dephosphorylation with calf intestinal phosphatase (NEB), and ligation of preadenylated 3' adapter (Universal micro RNA cloning primer, NEB). Ligated products were gel-eluted from a 12% TBE-urea gel and circularized using the Circligase single-stranded DNA ligase (Epicentre). Libraries were amplified using barcoded reverse primer and common forward primer and subjected to sequencing on the Illumina platform. Table 1 shows the reagents used for the experiments.

#### Mapping

After a quality check using the FastQC package, the fastq reads were mapped to the M. tb ribosomal RNA sequences using bowtie2. Unaligned reads were mapped to the M. tb genome downloaded from Ensembl using bowtie2. Resultant bam files were used for further analysis using in-house scripts available upon request.

#### Preprocessing

Genes shorter than 100nt in length were filtered out. Ignoring the first 21 nucleotides, the mean coverage scores for the remaining genes were calculated by summing up the total number of reads mapped per nucleotide and normalizing by the gene length. Only the genes with an average of 40 reads per nucleotide or above were taken for further analysis.

#### P-site offsets

We calculated p-site offsets for the read lengths ranging from 26 to 35 nt. For a particular length, reads mapping − 50 to + 200nt of an annotated initiation site were considered, and the number of 5′ (or 3′) termini aligning to each nucleotide position was noted. This information was represented as a vector of length 250 and normalized by dividing each element by its sum. Given similar vectors for M genes in our set, arranged as an M × 250 matrix, the metagene vector was obtained by a column-wise sum across the elements. These vectors were plotted, and the p-site offsets were subsequently determined by noting the extent of the shift of the initiation peak from the annotated start position.

#### Pause scores

For a gene g of length (in codons) Lg, the per-nucleotide read density vector (Rg) was split into chunks of three, corresponding to each codon (Cg1 … CgL). The first and last codons were ignored. A normalization factor (Fg) was calculated by taking the total sum of the read density vector divided by its length: Fg = sum(Rg)/(Lg-2). The positional pause score (Pgi) of the ith codon in the gene was computed by taking the sum of the vector-chunk associated with the codon (Cgi) and dividing it by the normalization factor: Pgi = sum(Cgi)/Fg. A codon's mean of positional pause scores across all genes was taken as its genomic pause score. For each amino acid, the pause scores were determined by taking the average of the genomic pause scores of its synonymous codons.

#### tRNA-HySeq

2 mg of total RNA from the H37Rv log phase culture was used for tRNA purification. Biological duplicates of RNA were used for library preparation, and quality was checked on a 1% formaldehyde agarose gel. Indeed, all the samples had suitable RNA quality in which 23S, 16SrRNAs, and the band containing shorter non-coding RNAs, including 5 s rRNA and tRNAs, are seen. To isolate tRNAs, total RNA was separated on 8% TBE-urea gel, and the bands corresponding to tRNAs based on size were cut and eluted. The eluted tRNA was partially digested with 0.1 M bicarbonate buffer, and the fragments were separated on a 15% TBE-urea gel. RNA oligo mix of size 19 nt and 35 nt was loaded along with the tRNA fragments for size selection. All RNA fragments coming between 19 and 35 nt were gel purified and ligated with a universal miRNA cloning linker (NEB). In subsequent steps, RNA markers were also ligated with adaptors to serve as size-selection markers. Adaptor-ligated tRNA fragments were separated from unligated ones on 12% TBE-urea gel. cDNA was synthesized from the purified tRNA fragments using Lib_RT_Rev primer, and cDNA was separated from the primer on 10% TBE-urea gel. Purified cDNA was circularized using Circligase and amplified using barcoded PCR primers. tRNA PCR products were separated on 8% Native gel; the lower bands indicated tRNA library products, while the upper band indicated adaptor-ligated RNA control. The libraries were sequenced on the Illumina HiSeq2500 machine.

### Calculation of TAc

TAc was calculated essentially as described previously (dos Reis, Savva, and Wernisch 2004)**.** The original formula used for the calculation of tRNA adaptability of codons was the following:

$${W}_{i}= {\sum }_{j=1}^{{n}_{i}}\left(1-{s}_{ij}\right){GCN}_{ij}$$

*W*_*i*_ is the absolute adaptiveness value of codon i; *n*_*i*_ is the number of isoacceptor tRNAs that can decode the codon i, *tGCN*_*ij*_ is the number of genes coding for the jth tRNA isoacceptor that can decode the codon i. *s*_*ij*_ is a measure of the strength of codon-anticodon interactions between the tRNA isoacceptor j and the codon i. If the base pairing is Watson–Crick, this value will be 0, but for the wobble base pairs, these are non-zero positive values. This value serves as a penalty for wobble base pairing. These values were calculated by dos Reis et al.^17^ and the same values were used in this study.

To incorporate tRNA abundance into the calculation, rather than the tRNA gene counts, we modified the equation as following:

$${W}_{i}= {\sum }_{j=1}^{{n}_{i}}\left(1-{s}_{ij}\right){tRNC}_{ij}$$

Here tRNC_ij_ indicates log2(tRNA read counts), which is considered as tRNA abundance. These absolute adaptiveness values were normalized to the highest Wi value across all 60 codons that code for an amino acid (methionine codon ATG was not used in this analysis). The TAc was calculated by the formula

$$TAc_{i} = \left\{ {\begin{array}{*{20}l} {W_{i} /W_{{Max~}} ,~} \hfill & {W_{i} ~ \ne 0~} \hfill \\ {W_{{mean~}} ,~} \hfill & {W_{i} = 0} \hfill \\ \end{array} } \right.~$$

where *TAc*_*i*_ is the relative adaptiveness value of codon i, *W*_*max*_ is the maximum of all *W*_*i*_ values, and *w*_*mean*_ is the geometric mean of all non-zero *w*_*i*_ values.

### Calculation of normalized codon usage, codon optimality and mRNA optimality values

Genomic codon usage was calculated using the equation given below.

$${cu}_{i} = \frac{\sum_{j=1}^{n}{C}_{ij}}{N}$$

$${CU}_{i}= \frac{{cu}_{i}}{{cu}_{max}}$$

where CU_i_ is the codon usage of *i*th codon, Cij is the number of *i*th codon in *j*th mRNA. N denotes the total number of codons in the genome.

To calculate the normalized codon usage, we calculated the codon count for each mRNA and these counts were multiplied with the normalized RNAseq counts of the mRNA. These values were used to estimate the abundance normalized codon usage.

$$n{cu}_{i} = \frac{\sum_{j=1}^{n}{C}_{ij}.{a}_{j}}{\sum_{i=1}^{m}\sum_{j=1}^{n}{C}_{ij}.{a}_{j}}$$

$${nCU}_{i}= \frac{{ncu}_{i}}{{ncu}_{max}}$$

Here, *nCUi* is the codon usage weighted for the mRNA abundance. *a*_*j*_ is the log2 transformed normalized read counts of mRNA *j* as estimated using RNAseq, and *m* is the number of codons.

To estimate the codon optimality the TAc values for each codon was divided by the nCU of that codon. To calculate the mOpt values, we multiplied the multiplied the number of occurrences of a codon in an RNA with the log2 transformed codon optimality values and added the resultant values for each codon. Finally, the optimality values obtained for each mRNA was normalized with the maximum optimality value across all mRNAs.

**Table 1:** Reagents used in this study. The source and catalog number are provided.

Reagents	Catalog no	Make
Middlebrook 7H9 Media	90,003–876	Difco middlebrook 7H9 Broth, BD Diagnostics
Chloramphenicol	CAS 56–75-7	Sigma-Aldrich
RNaseT1	LS01485	Worthington biochemical corporation
RNase I	AM2294	Thermo fisher scientific
S7 (Micrococcal) nuclease	LS04070	Worthington biochemical corporation
SUPERase.In	AM2694	Thermo fisher scientific
Trizol (RNAiso Plus)	9108	Takara bio USA
Calf intestine phosphatase (CIP)	MO290S	NEB
CircLigase II ssDNA ligase	CL4115K	Epicenter biotechnologies
Universal miRNA cloning linker	S1315S	NEB
Formaldehyde	F8775	Sigma-Aldrich

## Data Availability

The datasets supporting the conclusions of this article are available in the GEO repository (Accession No. GSE151718). Custom-made scripts used to analyze the ribosome profiling data are available on GitHub (https://github.com/Molecular-Genetics-Laboratory/bard).
